# ﻿Four new species of the spider genus *Synagelides* Strand, 1906 from South China (Araneae, Salticidae)

**DOI:** 10.3897/zookeys.1074.72823

**Published:** 2021-12-03

**Authors:** Bing Li, Cheng Wang,, Xian-Jin Peng

**Affiliations:** 1 College of Life Sciences, Hunan Normal University, Changsha, Hunan 410081, China Hunan Normal University Changsha China; 2 Guizhou Provincial Key Laboratory for Biodiversity Conservation and Utilization in the Fanjing Mountain Region, Tongren University, Tongren, Guizhou 554300, China Tongren University Tongren China

**Keywords:** Ant-like spider, jumping spider, taxonomy, Yunnan-Guizhou Plateau

## Abstract

Four new species of the jumping spider genus *Synagelides* Strand, 1906 from Guizhou and Yunnan, China are described: *Synagelidesangustus***sp. nov.** (♀), *S.latus***sp. nov.** (♂♀), *S.subagoriformis***sp. nov.** (♂♀), and *S.triangulus***sp. nov.** (♀). Photographs of the habitus and copulatory organs and a distributional map are provided.

## ﻿Introduction

Salticidae Blackwall, 1841, represented by 6345 species in 658 genera, is the most diverse spider family worldwide (World Spider Catalog 2021). *Synagelides* Strand, 1906 comprises a group of ant-like spiders which can be easily separated from all other salticid genera by having a triangular femoral apophysis and an inflated patella of male palp (Peng 2020). *Synagelides* species are distributed mostly in Asia, from the Far East of Russia to Southeast Asia (Yin et al. 2012; Peng 2020; Wang et al. 2020). In the last 10 years, a series of studies (Barrion et al. 2013; Caleb et al. 2018; Kanesharatnam and Benjamin 2020; Lin and Li 2020; Liu et al. 2017; Logunov 2017; Wang et al. 2020) have resulted in the description of 20 new species and increased the total species number of the genus to 57, of which including 30 from China (World Spider Catalog 2021).

Recently, while examining spider specimens collected from the Yunnan-Guizhou Plateau, four species of the genus *Synagelides* were identified as new to science and are described here.

## ﻿Material and methods

The specimens were collected mainly by beating shrubs and screening leaf litter. All specimens were preserved in 75% ethanol and are deposited in the museum of Tongren University (TRU), Tongren, China. The specimens were examined with an Olympus SZ51 stereomicroscope. Epigynums were cleared in Trypsin enzyme solution before examination and imaging. Left male palps, legs I, and chelicerae were used for illustration. Photographs were taken with a Kuy Nice CCD mounted on an Olympus BX51 compound microscope. Compound focus images were generated using Helicon Focus v. 6.7.1 software. All measurements are given in millimeters. Leg measurements are given as: total length (femur, patella + tibia, metatarsus, tarsus). References to figures in the literature are listed in lowercase type (fig. or figs); figures in this paper are noted with an initial capital (Fig. or Figs). Terminology follows Lin and Li (2020). Abbreviations used in the text and figures are as follows:

**ALE** anterior lateral eye

**AME** anterior median eye

**AR** atrial ridge

**BTA** basal tibial apophysis

**CD** copulatory duct

**CO** copulatory opening

**DCA** dorsal cymbial apophysis

**E** embolus

**EFL** eye field length

**F** fold

**FD** fertilization duct

**GD** gland duct

**H** hood

**MA** median apophysis

**MS** median septum

**PCA** prolateral cymbial apophysis

**PERW** posterior eye row width

**PLE** posterior lateral eye

**RTA** retrolateral tibial apophysis

**S** spermatheca

**SD** sperm duct

## ﻿Taxonomy

### Family Salticidae Blackwall, 1841

**Genus *Synagelides* Strand, 1906**

#### ﻿ Synagelides angustus

Taxon classificationAnimaliaAraneaeSalticidae

Wang, Li & Peng sp. nov.

B63000BF-C740-5EC4-B223-73CC499BDC53

http://zoobank.org/1D33F837-DD2D-44C9-B7C1-DD140C6CBAE7

Figs 1 , 7

##### Type material.

***Holotype*.** ♀ (TRU-JS 651): China: Guizhou Province: Jiangkou County: Dewang Township: Baxi Village, 27°51.68'N, 108°36.88'E, elevation: 897 m, 15.VI.2015, P. Luo, X. Kuang, G. Liu, T. Liu, Z. Liao, M. Liao and C. Wang leg. ***Paratype*.** 1♀ (TRU-JS 652), same locality as holotype, 13.VII.2013, X. Mi and M. Liao leg.

##### Etymology.

The specific name is from the Latin “*angustus*” and refers to the long and narrow epigynal median septum; adjective.

##### Diagnosis.

**Figure 1.**
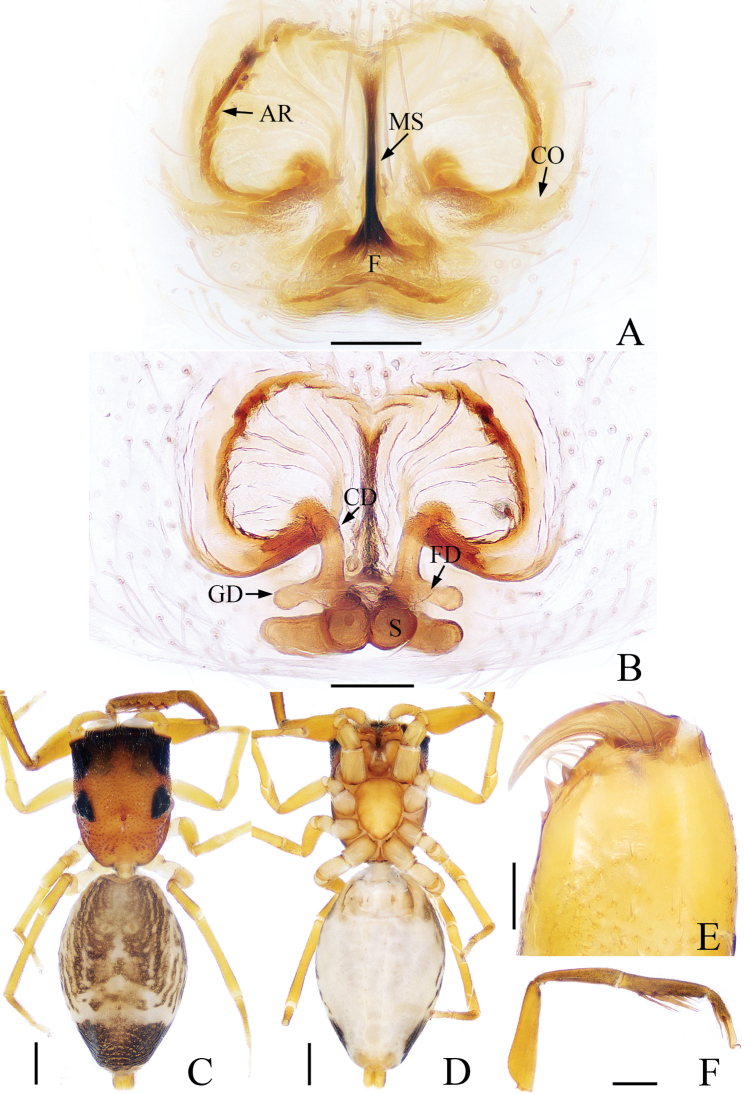
*Synagelidesangustus* sp. nov., holotype. **A** epigynum, ventral view **B** internal genitalia, dorsal view **C** habitus, dorsal view **D** habitus, ventral view **E** chelicera, posterior view **F** leg I, retrolateral view. Scale bars: 0.1 mm **A, B, E**; 0.5 mm **C, D, F**

*Synagelidesangustus* sp. nov. resembles *S.subgambosus*Wang et al. 2020 in having a long and narrow epigynal median septum, a pair of arc-shaped atrial ridges, and a posteriorly located epigynal fold. However, *S.angustus* sp. nov. can be distinguished from *S.subgambosus* by the following characters: 1) posterior margin of epigynal fold arc-shaped (Fig. 1A) in *S.angustus* sp. nov., but straight in *S.subgambosus* (fig. 12A in Wang et al. 2020); 2) median septum 20 times longer than wide in *S.angustus* sp. nov. (Fig. 1A), but about six times longer than wide in *S.subgambosus* (fig. 12A in Wang et al. 2020).

##### Description.

**Female** (holotype). Total length 4.06. Carapace 1.59 long, 1.19 wide. Abdomen 2.41 long, 1.55 wide. Eye sizes and interdistances: AME: 0.40, ALE: 0.22, PLE: 0.21, AREW: 1.22, PERW: 1.22, EFL: 1.01. Leg measurements: I: 4.07 (1.27, 2.07, 0.41, 0.32); II: 2.75 (0.85, 1.02, 0.56, 0.32); III: 3.00 (0.85, 1.02, 0.76, 0.37); IV: 4.28 (1.17, 1.61, 1.10, 0.40). Carapace (Fig. 1C) stippled, reddish-brown, darker anteriorly with brown spots, covered with white hairs anteriorly and laterally. Fovea oval, hollowed. Chelicerae (Fig. 1E) yellow, with two promarginal teeth and one retromarginal fissidentate tooth. Endites and labium (Fig. 1D) yellow, lighter anteriorly, covered with thin brown hairs. Sternum (Fig. 1D) yellow, scutiform, lighter postero-medially, covered with short, thin hairs. Legs yellow except patellae and metatarsi I brown, legs I (Fig. 1F) with five pairs of ventral spines on tibia and two pairs of ventral spines on metatarsus. Abdomen (Fig. 1C, D) ovoid, dorsum brown, darker posteriorly, median area with two pairs of apodemes, posterior area with a wide, irregular horizontal white stripe; venter grayish-white. Epigynum (Fig. 1A, B): almost as long as wide, with a pair of lateral arc-shaped ridges; atrium large, separated by a narrow median septum; copulatory openings located posteriorly; copulatory ducts extending upwards obliquely and then descending posteriorly along longitudinal axis, basally with short gland ducts; spermathecae elongated, extending horizontally; fertilization ducts lamellar.

**Male.** Unknown.

##### Distribution.

Guizhou Province, China (Fig. 7).

#### ﻿ Synagelides latus

Taxon classificationAnimaliaAraneaeSalticidae

Wang, Li & Peng sp. nov.

046CFBB5-2A4F-54AB-AF01-A2C52C147350

http://zoobank.org/8084B2A4-E509-4839-B7A1-88B021EF6B28

Figs 2 , 3 , 7

##### Type material.

***Holotype*.** ♂ (TRU-JS 653): China: Yunnan Province: Nanjian County: Baohua Town: A’pa Village, 24°51.60'N, 100°26.00'E, elevation: 2310 m, 11.VIII.2015, C. Wang, Z. Liao, P. Luo and G. Liu leg. ***Paratype*.** 1♀ (TRU-JS 654), same date as the holotype.

##### Etymology.

The specific name is from the Latin “*latus*”, and refers to the wide basal tibial apophysis; adjective.

##### Diagnosis.

**Figure 2.**
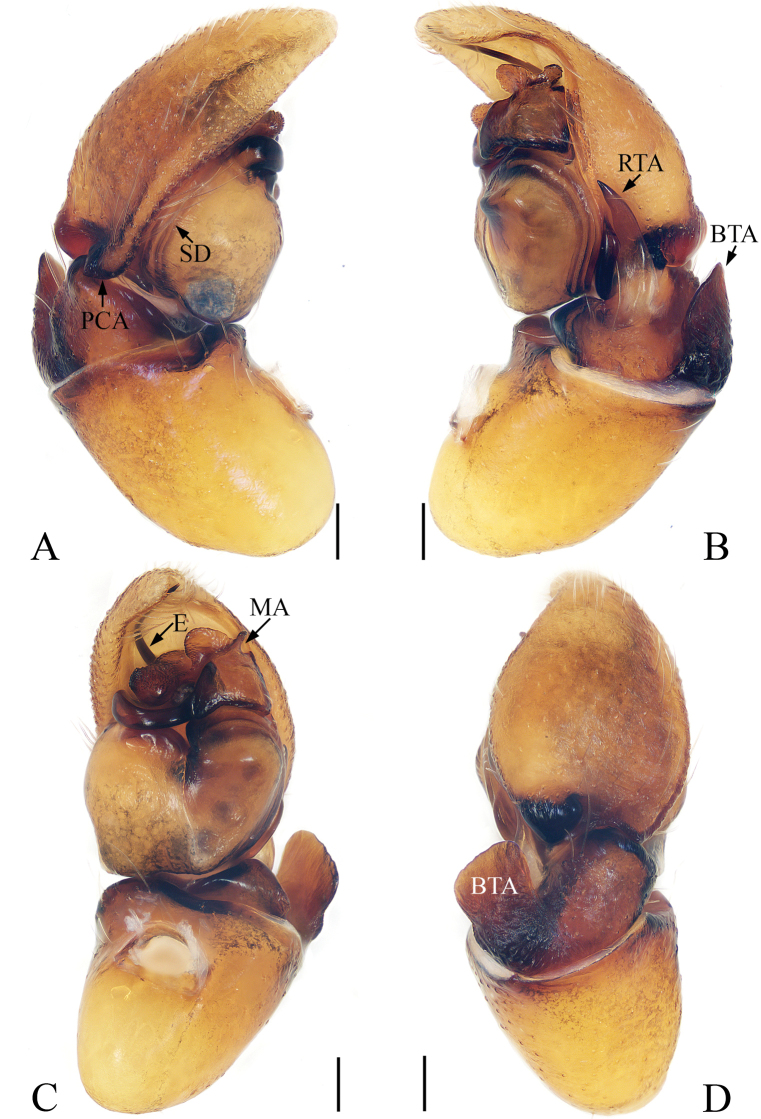
Left male palp of *Synagelideslatus* sp. nov., holotype. **A** prolateral view **B** retrolateral view **C** ventral view **D** dorsal view. Scale bars: 0.1 mm **A–D**.

**Figure 3.**
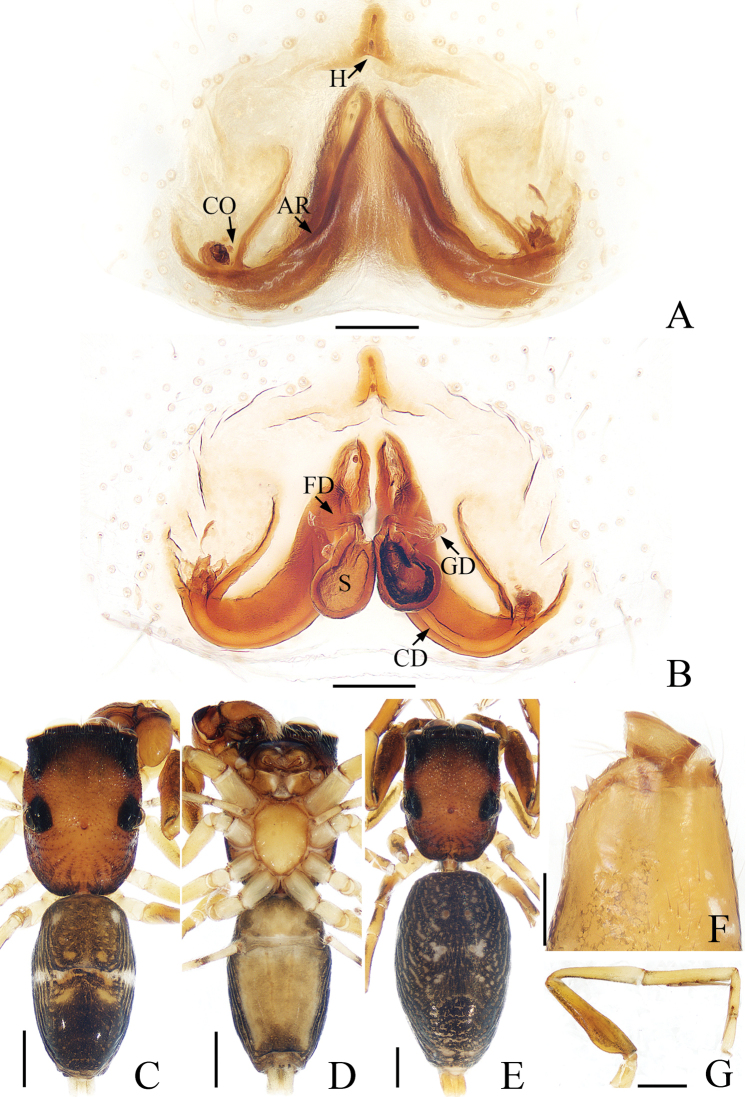
*Synagelideslatus* sp. nov. **A** epigynum, ventral view **B** internal genitalia, dorsal view **C** holotype habitus, dorsal view **D** holotype habitus, ventral view **E** paratype habitus, dorsal view **F** holotype chelicera, posterior view **G** holotype leg I, prolateral view. Scale bars: 0.1 mm **A, B, F**; 0.5 mm **C–E, G**.

*Synagelideslatus* sp. nov. resembles *S.wuliangensis*Wang et al. 2020 in having two tibial apophyses and an anterior epigynal hood, but it differs from *S.wuliangensis* by the following characters: 1) RTA about 1/4 cymbial length in *S.latus* sp. nov. (Fig. 2B), but almost 1/2 in *S.wuliangensis* (fig. 13B in Wang et al. 2020); 2) BTA as long as wide in dorsal view in *S.latus* sp. nov. (Fig. 2D), whereas longer than wide in *S.wuliangensis* (second retrolateral tibial apophysis in fig. 13F in Wang et al. 2020); 3) tibia of leg I with four pairs of spines in *S.latus* sp. nov. (Fig. 3G), whereas five pairs in *S.wuliangensis* (fig. 14H in Wang et al. 2020); 4) distance between epigynal hood and tip of median septum much shorter than median septum in *S.latus* sp. nov. (Fig. 3A), but much longer than median septum in *S.wuliangensis* (fig. 14A in Wang et al. 2020).

##### Description.

**Male** (holotype). Total length 2.93. Carapace 1.24 long, 1.03 wide. Abdomen 1.56 long, 0.89 wide. Eye sizes and interdistances: AME: 0.33, ALE: 0.19, PLE: 0.18, AREW: 0.99, PERW: 1.08, EFL: 0.88. Leg measurements: I: 4.59 (1.39, 2.32, 0.56, 0.32); II: 2.47 (0.73, 0.88, 0.54, 0.32); III: 2.64 (0.76, 0.90, 0.66, 0.32); IV: 3.32 (0.95, 1.27, 0.78, 0.32). Carapace (Fig. 3C) stippled, covered with sparse and thin hairs anteriorly. Eye base black. Fovea oval, hollowed, cervical and radial groove indistinct. Chelicerae (Fig. 3F) yellow, with two promarginal teeth and one retromarginal fissidentate tooth. Endites (Fig. 3D) as long as wide, lighter antero-internally. Labium (Fig. 3D) brown except white basally, covered with sparse black hairs. Sternum (Fig. 3D) yellow, scutiform. Legs I (Fig. 3G) with four pairs of ventral spines on tibia, two pairs of ventral spines on metatarsus. Abdomen (Fig. 3C, D) oblong, dorsum brown, darker posteriorly, apodemes indistinct, with a pair of round white spots on anterior edge, a horizontal stripe of white hairs and two pairs of irregular yellow spots in median area, several arc-shaped lines of spots in posterior area; venter grayish-brown, covered with dark-brown spots posteriorly. Palp (Fig. 2A–D): patella swollen; tibia stubby, RTA sword-shaped, PCA wide, shovel-shaped; bulb big, separated by crevice; embolus spiraling, tip reaching cymbial apex; median apophysis complicated and sclerotized.

**Female** (paratype). Total length 4.30. Carapace 1.65 long, 1.22 wide. Abdomen 2.56 long, 1.44 wide. Eye sizes and interdistances: AME: 0.40, ALE: 0.22, PLE: 0.21, AREW: 1.18, PERW: 1.28, EFL: 1.10. Leg measurements: I: 4.03 (1.27, 1.90, 0.49, 0.37); II: 2.75 (0.85, 1.02, 0.56, 0.32); III: 2.98 (0.88, 1.02, 0.76, 0.32); IV: 4.05 (1.15, 1.51, 1.02, 0.37). Habitus (Fig. 3E) similar to those of male except two pairs of apodemes distinct and with a pair of irregular white spots instead of the horizontal stripe in middle of dark-brown abdomen. Epigynum (Fig. 3A, B): wider than long, hood narrow, bell-shaped; atrium large, with a pair of arc-shaped ridges; copulatory openings situated postero-laterally; copulatory ducts stout, eggplant-shaped, gland ducts present; spermathecae pear-shaped, touching each other anteriorly; fertilization ducts lamellar, extending horizontally.

##### Distribution.

Yunnan Province, China (Fig. 7).

#### ﻿ Synagelides subagoriformis

Taxon classificationAnimaliaAraneaeSalticidae

Wang, Li & Peng sp. nov.

D9388574-44B0-5441-B28E-BC9EDEC53E6E

http://zoobank.org/5C453648-6ACD-4C79-BFFE-941038038AA9

Figs 4 , 5 , 7

##### Type material.

***Holotype*.** ♂ (TRU-JS 655): China: Guizhou Province: Tongren City: Shiqian County: Ganxi Town: Fuyan Village, 27°21.46'N, 108°20.26'E, elevation: 859 m, 28–30.IV.2017, X. Mi, C. Wang, Y. Mi, S. Lei, G. Tian and H. Liu leg. ***Paratypes*.** 2♀♀ (TRU-JS 656–657), China: Guizhou Province: Tongren City: Shiqian County: Pingshan Town: Fodingshan Village, 27°21.50'N, 108°09.35'E, elevation: 859 m, 12.VII.2017, X. Mi, C. Wang, G. Tian and H. Liu leg.; 1♀ (TRU-JS 658), same locality as the holotype, 27°21.65'N, 108°01.98'E, elevation: 708 m, 16.VII.2017, X. Mi, C. Wang, F. Li, G. Tian and H. Liu leg.; 4♂♂, 3♀♀ (TRU-JS 659–665), same date as the holotype.

##### Etymology.

The specific name is from its similarity to *S.agoriformis* Strand, 1906; substantive.

##### Diagnosis.

**Figure 4.**
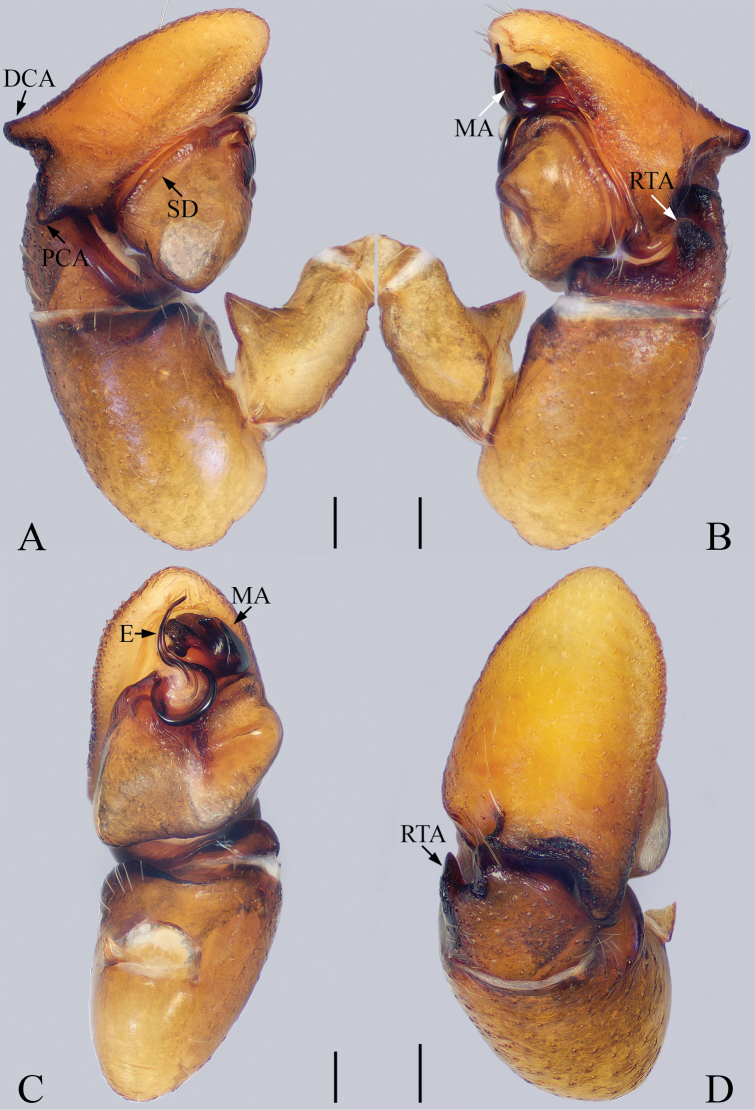
Left male palp of *Synagelidessubagoriformis* sp. nov., holotype. **A** prolateral view **B** retrolateral view **C** ventral view **D** dorsal view. Scale bars: 0.1 mm **A–D**.

**Figure 5.**
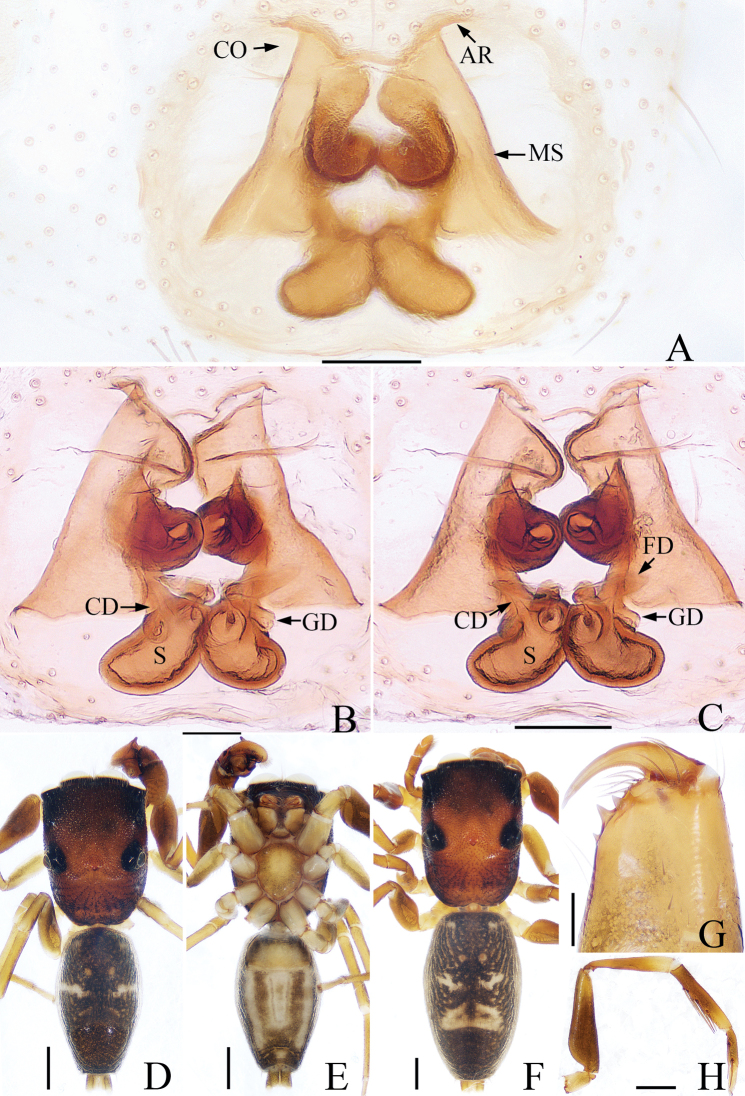
*Synagelidessubagoriformis* sp. nov. **A** epigynum, ventral view **B, C** internal genitalia, dorsal view **D** holotype habitus, dorsal view **E** holotype habitus, ventral view **F** female paratype habitus, dorsal view **G** holotype chelicera, posterior view **H** holotype leg I, prolateral view. Scale bars: 0.1 mm **A–C, G**; 0.5 mm **D–F, H**.

*Synagelidessubagoriformis* sp. nov. most closely resembles *S.agoriformis* Strand, 1906, but it differs from *S.agoriformis* by the following characters: 1) RTA present in *S.subagoriformis* sp. nov. (Fig. 4B, D), whereas absent in *S.agoriformis*; 2) the length of genital bulb more than 2/3 cymbial length in *S.subagoriformis* sp. nov. (Fig. 4B) whereas about 1/2 cymbial length in *S.agoriformis* (fig. 20 in Omelko and Fomichev 2021); 3) median septum as long as wide in *S.subagoriformis* sp. nov. (Fig. 5A), whereas wider than long in *S.agoriformis* (figs 29–32 in Omelko and Fomichev 2021); 4) spermathecae extending obliquely in *S.subagoriformis* sp. nov. (Fig. 5B, C), whereas extending horizontally in *S.agoriformis* (figs 33, 34 in Omelko and Fomichev 2021).

##### Description.

**Male** (holotype). Total length 3.44. Carapace 1.67 long, 1.22 wide. Abdomen 1.75 long, 1.01 wide. Eye sizes and interdistances: AME: 0.41, ALE: 0.24, PLE: 0.21, AREW: 1.24, PERW: 1.21, EFL: 1.01. Leg measurements: I: 4.37 (1.34, 2.20, 0.49, 0.34); II: 2.72 (0.83, 0.98, 0.59, 0.32); III: 2.87 (0.85, 1.00, 0.68, 0.34); IV: 3.87 (1.07, 1.46, 0.93, 0.41). Carapace (Fig. 5D) reddish-brown, darker anteriorly, covered with thin hairs. Fovea oval, hollowed. Chelicerae (Fig. 5G) yellow, with two promarginal teeth and one retromarginal fissidentate tooth with two cusps. Endites (Fig. 5E) longer than wide, white medially, covered with brown hairs. Labium (Fig. 5E) yellowish-brown, anteriorly covered with thin hairs. Sternum (Fig. 5E) scutiform, lighter medially. Legs I (Fig. 5H) with three pairs of ventral spines on tibia and two pairs of ventral spines on metatarsus. Abdomen (Fig. 5D, E) oblong, dorsum dark-brown, a pair of grayish-white spots in anterior area, one discontinuous white horizontal stripe and two pairs of apodemes in median area, two indistinct herringbone stripes in posterior area; venter grayish-white, with a pair of brown longitudinal stripes in bilateral areas, covered with dark-brown spots in posterior area. Palp (Fig. 4A–D): patella swollen, longer than wide; tibia stubby, RTA sclerotized, finger-shaped; cymbium with dorsal and prolateral apophyses; bulb swollen; embolus flat, basal portion semicircular, distal portion thin, bent and blunt; median apophysis sclerotized, with little tubercles.

**Female** (paratype, TRU-JS 656). Total length 4.96. Carapace 2.22 long, 1.59 wide. Abdomen 2.67 long, 1.57 wide. Eye sizes and interdistances: AME: 0.52, ALE: 0.28, PLE: 0.26, AREW: 1.61, PERW: 1.62, EFL: 1.33. Leg measurements: I: 4.99 (1.54, 2.54, 0.54, 0.37); II: 3.49 (1.10, 1.29, 0.73, 0.37); III: 3.76 (1.10, 1.34, 0.95, 0.37); IV: 5.25 (1.46, 2.01, 1.32, 0.46). Habitus (Fig. 5F) similar to those of males except white horizontal stripe in median area shorter, and one white stripe whose shape near triangular contour in posterior area of abdomen. Epigynum (Fig. 5A–C): atrial ridges located along front margin of epigynum, roughly bow-shaped; median septum trapezoidal, wider basally; copulatory openings below the lateral sides the atrial ridges; copulatory ducts extending upward, distal portion coiled, with short gland ducts; spermathecae touching each other anteriorly; fertilization ducts lamellar, originating from top of inner sides of spermathecae, extending horizontally.

##### Distribution.

Guizhou Province, China (Fig. 7).

#### ﻿ Synagelides triangulus

Taxon classificationAnimaliaAraneaeSalticidae

Wang, Li & Peng sp. nov.

85CF9336-A34F-5257-AD8F-73877B514893

http://zoobank.org/5F8655C0-5BA8-4A79-BC3C-CF278FB013C6

Figs 6 , 7

##### Type material.

***Holotype*.** ♀ (TRU-JS 666): China: Yunnan Province: Kunming City: Xishan Forest Park: 24°59.00'N, 102°37.01'E, elevation: 2117 m, 9.VIII.2015, C. X. Mi, C. Wang, M. Liao, Z. Liao, P. Luo, X. Kuang, T. Liu and G. Liu leg. ***Paratypes*.** 1♀ (TRU-JS 667), same locality as the holotype, 13.VII.2013; 1♀ (TRU-JS 668), same locality as the holotype, 16.VIII.2018, C. Wang, H. Liu and Y. Yang leg.

##### Etymology.

The specific name is from the Latin “*triangulus*” and refers to the triangular epigynal hood; adjective.

##### Diagnosis.

**Figure 6.**
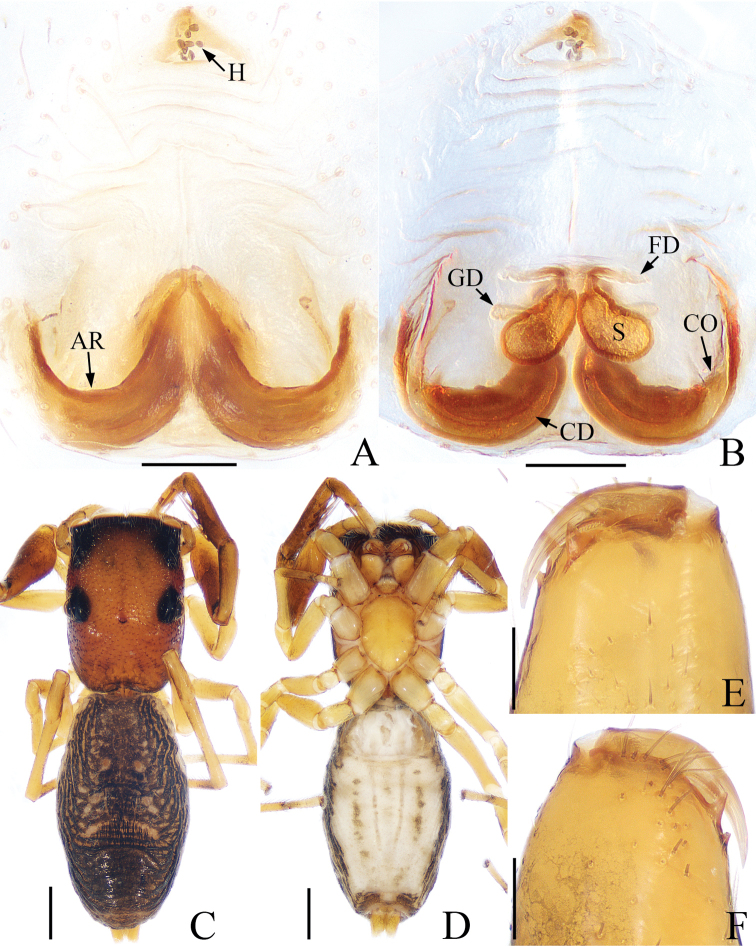
*Synagelidestriangulus* sp. nov., holotype. **A** epigynum, ventral view **B** internal genitalia, dorsal view **C** habitus, dorsal view **D** habitus, ventral view **E** chelicera, posterior view **F** chelicera, anterior view. Scale bars: 0.1 mm **A, B, E, F**; 0.5 mm **C, D**.

*Synagelidestriangulus* sp. nov. most closely resembles *S.hamatus*Zhu et al. 2005 in having the epigynal hood far away from the atrial ridge, but it differs from *S.hamatus* by the following characters: 1) atrial ridges horizontally arc-shaped in *S.triangulus* sp. nov. (Fig. 6A), whereas longitudinally arc-shaped in *S.hamatus* (fig. 12B in Zhu et al. 2005); 2) spermathecae medially located in *S.triangulus* sp. nov. (Fig. 6B) but posteriorly located in *S.hamatus* (fig. 12C in Zhu et al. 2005).

##### Description.

**Female** (holotype). Total length 5.68. Carapace 2.36 long, 1.68 wide. Abdomen 3.27 long, 1.86 wide. Eye sizes and interdistances: AME: 0.35, ALE: 0.19, PLE: 0.18, AREW: 1.06, PERW: 1.16, EFL: 0.92. Leg measurements: I: 3.44 (1.05, 1.61, 0.46, 0.32); II: 2.53 (0.78, 0.95, 0.51, 0.29); III: 2.59 (0.73, 0.93, 0.61, 0.32); IV: 3.73 (1.07, 1.44, 0.88, 0.34). Carapace (Fig. 6C) stippled, reddish-brown. Eye base black. Fovea oval, hollowed, cervical and radial groove indistinct. Chelicerae (Fig. 6E, F) yellow, with two promarginal teeth and one retromarginal fissidentate tooth. Endites and labium (Fig. 6D) yellow, lighter anteriorly, covered with dark thin hairs. Sternum (Fig. 6D) yellow, scutiform. Legs yellow except legs I reddish-brown, legs I (Fig. 6C, D) with three pairs of ventral spines on tibia, two pairs of ventral spines on metatarsus. Abdomen (Fig. 6C, D) oblong, dorsum dark brown, two pairs of spots, two pairs of apodemes and several indistinct herringbone stripes in median area; venter grayish-white, with two lines of spots and two grayish-brown longitudinal stripes of spots. Epigynum (Fig. 6A, B): longer than wide, with wrinkles under triangular epigynal hood; atrial ridges horizontal arc-shaped; copulatory openings located postero-laterally, indistinct; copulatory ducts long, main portion arc-shaped and extending horizontally; spermathecae pear-shaped, touching each other anteriorly; fertilization ducts extending horizontally.

**Male.** Unknown.

##### Distribution.

**Figure 7.**
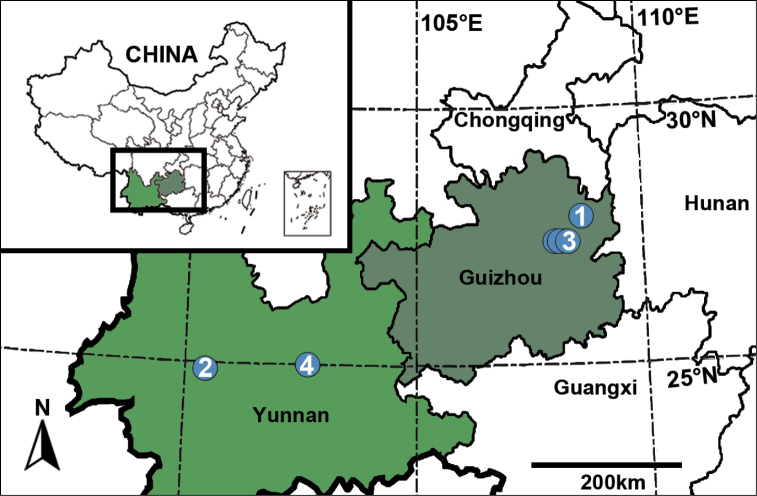
Type localities of new species of *Synagelides*. **1***S.angustus* sp. nov. **2***S.latus* sp. nov. **3***S.subagoriformis* sp. nov. **4***S.triangulus* sp. nov.

Yunnan Province, China (Fig. 7).

## Supplementary Material

XML Treatment for Synagelides angustus

XML Treatment for Synagelides latus

XML Treatment for Synagelides subagoriformis

XML Treatment for Synagelides triangulus
